# Integrating Ethical Principles Into the Regulation of AI-Driven Medical Software

**DOI:** 10.7759/cureus.79506

**Published:** 2025-02-23

**Authors:** Filzah Faheem, Mahdi Haq, Mohamed Derhab, Reeda Saeed, Usman Ahmad, Junaid S Kalia

**Affiliations:** 1 Neurology, NeuroCare.AI, Dallas, USA; 2 Neurology, Mayo Clinic, Rochester, USA

**Keywords:** artificial intelligence, deep learning, ethics, machine learning, medical devices, regulations

## Abstract

In recent years, a sharp increase in artificial intelligence (AI)-based software as medical devices has been seen in the United States and the European Union. Despite the huge potential of these devices in alleviating suffering through rapid identification and early intervention, their adoption in clinical practice has remained relatively slow due to ethical questions surrounding their usage. Even though there is no universal framework for the approval of these devices, the guiding principles behind individual regulatory bodies almost stay the same, with some more focused on the technical aspect while others involving the ethical aspects as well. The International Medical Device Regulators Forum devised a SaMD Working Group to outline the essential controls guiding the approval of these devices, but there is a lack of a structured approach for the regulatory approval process. This article outlines the principles of medical ethics, such as autonomy, beneficence, and fair distribution of healthcare sources, and how they relate to the use of AI-based devices. The core regulatory guidelines are then viewed in light of these ethical principles. We recommend that a comprehensive regulatory framework with integration of principles of medical ethics be made public. Though no universally accepted framework is available, regulating quality management, risk assessment, and data privacy would help build trust to promote the adoption of AI in healthcare.

## Introduction and background

Landscape of medical ethics

The domain of medical ethics is as old as the field of medicine itself and is under continuous evolution. A significant milestone in the field of ethics was achieved in 1803 when British physician Thomas Percival published his work “Medical Ethics,” thus formally coining the term [1] and laying the foundation for ethical considerations in modern medical practice [2]. Potter in 1970 expanded the scope of medical ethics when he used the term “bioethics” [3] and changed the way of looking at medical ethics from the Hippocratic paternalism lens to a more suitable approach encompassing autonomy, beneficence, consent, and non-maleficence [4]. The experts are actively engaged in exploring the ethical implications of new emerging technologies and developing guidelines to ensure their ethical and responsible implementations [5].

With the rapid ongoing digital transformation of every field of life, the integration of artificial intelligence (AI) in medicine brings forth a whole new set of ethical challenges that need to be addressed [6]. Although this confluence of two highly advanced disciplines has the potential to revolutionize medical practice and enhance the efficiency of healthcare systems [7], it has also raised ethical concerns regarding the use of AI-based devices in clinical settings. Explainability and transparency of AI algorithms are the characteristics that are crucial to ensuring the trust and accountability of these systems. Ethical frameworks are often taken as guiding principles to assess the ethical challenges associated with AI in medicine [6], but a more structured regulatory framework integrating the ethical component is essential to ensure patient safety.

Software as a medical device and artificial intelligence: key definitions

The world saw a rapid development of AI-based systems in various disciplines, ranging from agriculture to finance, in recent years. “Software as a medical device” or SaMD is one of the emerging AI-based products that has a lot of ethical concerns associated with their use but a less structured approval pathway to address them [8,9]. There are multiple definitions of SaMD, but the “International Medical Device Regulators Forum” defines it as “software intended to be used for one or more medical purposes that perform these purposes without being part of a hardware medical device” [10]. SaMD has the potential to revolutionize healthcare by leveraging AI to improve patient care [11]. John McCarthy, who coined the term AI in 1955, defined it as “the science and engineering of making intelligent machines” [12]. Machine learning (ML) is a branch of AI that makes computer systems capable of learning without being explicitly programmed by the use of algorithms trained on specific data [13]. Deep learning is a subset of ML that employs the power of artificial neural networks containing multiple layers to learn and make predictions about highly complex data [14]. SaMD employs the power of these AI algorithms to serve a vast variety of diagnostic and predictive functions.

Problem statement

Due to being an emerging discipline, the current research focus is mainly on the development of a universal and well-accepted regulatory framework that somewhat overlooks the ethical aspects related to SaMD use. The rapid approval rate of low-risk SaMD can further turn a blind eye to this aspect and can compromise it [15]. In this article, we aim to explore what ethical concerns SaMD raises and if the existing regulatory approval process sufficiently addresses those issues. The regulatory controls, as proposed by the working group of the International Medical Device Regulation Forum, are taken as references to evaluate if they help in addressing various ethical issues of SaMD [16].

With the use of AI-enabled models, the concerns about ethical principles increase such as the use of AI in patients adversely affected with psychiatric disorders might not be in their benefit because these patients need human-human interaction and compassion. Similarly, the model used by IBM Watson to assess the information and medical data of cancer patients was criticized due to unsafe recommendations for cancer treatment, though no unsafe recommended treatment was given to real patients [17,18]. The algorithms trained on biased data also raise concerns regarding decisions made using that data on different populations [19]. In healthcare, the doctor-patient relationship is based on empathy and sympathy. Though AI in healthcare is advancing, AI applications or robots will have difficulty in developing empathy behaviour towards patients, and patients might not have appropriate behaviour when dealing with robot physicians [18].

Search methodology

Using PubMed and Scopus databases, all articles related to AI and medical ethics were reviewed. Among the keywords used to search the database were “SaMD”, “Software Medical Devices Regulations”, “Regulatory approval”, “Medical ethics”, and “bioethics”. Since this is a narrative review, no statistical methods were used. Also, no specific inclusion or exclusion criteria were implemented.

## Review

Principles of medical ethics in the context of AI-based SaMD

**Figure 1:**
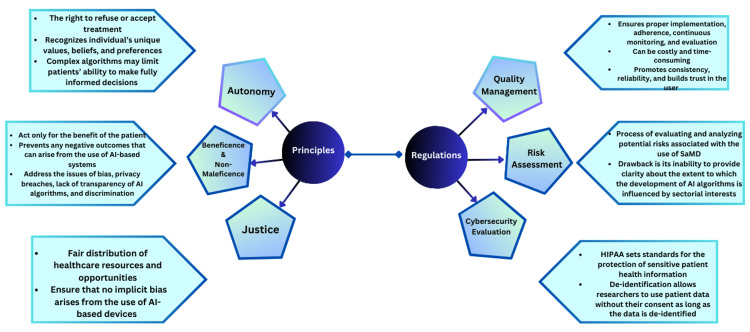
Principles and regulatory framework of artificial intelligence The figure was created by the authors.

AI’s potential to significantly impact various disciplines gave rise to a call for action for the development of guidelines for its usage [20]. The potential of AI-based devices on human jobs is an area of concern where many fear that AI and automation can bring about significant advancement leading to job displacement for human workers [21,22]. Younger individuals with low levels of education are more susceptible to such job displacements [23]. Fear of use by malevolent actors [24] and concerns regarding discrimination against certain population groups [25] are areas of huge debate. A framework of principles of medical ethics as proposed by Beauchamp and Childress can be used in the ethical analysis of AI-based systems and guide ethical decision-making. Different national and international organizations are continuously working on the development of ethics guidelines for AI to address these concerns [26]. In the subsequent section, the ethical issues arising from the use of SaMD are discussed, with a focus on a more traditional ethical framework consisting of autonomy, non-maleficence, beneficence, and justice (Figure 1).

Autonomy

The principle of autonomy gives patients the right to make decisions about their healthcare and expects that they should be respected. It puts special emphasis on the importance of informed consent, self-determination, and the right to refuse or accept treatment [27]. The ethics guidelines from different organizations include various terms that fall into the domain of autonomy, such as freedom of expression, empowerment, positive freedom, freedom to flourish, freedom to withdraw consent, and freedom from manipulation and technological experimentation [28]. All these terms serve the same purpose - to give the patient full control over the decision-making process about his health. Autonomy recognizes that individuals have unique values, beliefs, and preferences that guide their healthcare decisions and that they must be respected. SaMDs may interfere with the principle of autonomy due to their inherently complex algorithms for the decision-making process. These complex algorithms may generate predictions that are difficult to understand and can limit patients’ ability to make fully informed decisions about their health. The heavy reliance of healthcare providers on the output of AI-based systems can also undermine patient autonomy as patients might feel obliged to comply with the recommendations of technology while ignoring their preferences [29]. The notion of patient privacy and confidentiality also falls in this domain. For data-hungry ML and deep-learning algorithms, a vast amount of medical data is required for training purposes. The question of informed consent and data ownership is a complex one and varies with region. The European General Data Privacy Protection Law gives users data ownership, and users can decide how their data can be used; however, rules are different in the United States regarding data ownership [30].

Beneficence and Non-maleficence

The concepts of beneficence and non-maleficence can be traced back to the era of Hippocrates [31] and are two fundamental principles of medical ethics that are complementary to each other (“do good and do not harm”). Beneficence obliges the physician to act only for the benefit of the patient and avoid anything that could oppose the patient’s well-being [32]. The principle of non-maleficence encompasses a whole set of rules that prevents physicians from harming patients in any capacity in any way [31]. Non-maleficence is also a principle of social justice as it aligns with broader societal concerns of fairness, equity, and well-being of vulnerable populations [33]. AI-powered clinical decision support systems can analyze large amounts of patient data to provide more accurate diagnoses and treatment plans to healthcare providers. They comply with the principle of beneficence as they improve the accuracy and effectiveness of the healthcare system and contribute to better patient outcomes [6]. This principle can also be seen in action in the use of AI in predictive analytics for screening patients at risk of developing a specific health condition. By identifying patterns and risk factors in patient data that would otherwise be unrecognizable by physicians, these AI-based devices lead to timely intervention and preventive measures [34].

Non-maleficence prevents any negative outcomes that can arise from the use of AI-based systems. This principle calls for the need to address the issues of bias, privacy breaches, lack of transparency of AI algorithms, and discrimination [35,36]. It is interesting to know that nonmaleficence is addressed more in literature than its counterpart beneficence, which indicates the priority of AI researchers to avoid harm at all costs [28].

Justice

Justice in medical ethics refers to a fair distribution of healthcare resources and opportunities and involves the provision of services to individuals fairly and equitably [26,37]. The term “justice” in the context of AI refers to fairness, diversity, equality, inclusion, accessibility, elimination of bias and discrimination, and the right to appeal decisions [28]. Appropriate measures must be taken to ensure that no implicit bias arises from the use of AI-based devices and that unfair discrimination is eliminated during the development process [38]. When AI algorithms are trained on biased datasets, they can perpetuate and even amplify existing biases, leading to discriminatory and unfair outcomes. An example of such discrimination was revealed in a study by Obermeyer et al., in which an AI algorithm to allocate healthcare resources was found to be discriminatory against African American patients. He explained that the bias exists in these algorithms because these algorithms are more focused on healthcare costs rather than the illnesses with which patients present [39]. Topol gives many examples of data bias, including datasets used in dermatology to diagnose melanoma without including skin color, and using genomic data compiled in the United States where minorities are underrepresented. Therefore, excluding minorities from the data sets leads to prejudice in the AL algorithms [40]. Such incidents can be avoided by employing a robust and carefully planned data collection process. By ensuring that datasets used for training AI algorithms are diverse and represent the diversity of the population they aim to serve, the risk of algorithmic bias can be mitigated [41]. The AI ethics guidelines suggest multiple measures to promote justice, ranging from the development of technical solutions to strengthening government oversight [28].

Current regulatory framework for AI-based SaMD

There is currently no universally accepted framework for AI-based SaMD approval [42]. Regulatory bodies around the world have their own guidelines to regulate SaMD, which takes their root from existing frameworks for medical device approval. In the United States, the FDA is the main organization responsible for evaluating and approving SaMD intended for diagnostic, treatment, and preventive purposes [43]. Challenges remain in the approval and regulation process of SaMD due to the rapidly evolving nature of AI algorithms, and a distinct and more structured regulatory framework is required to eliminate the risk of racial disparities and bias that can be caused by these [44,45]. In the European Union, the European Medicines Agency (EMA) regulates the SaMD according to the risk level and has a regulatory framework in action for AI and ML-based medical devices [46]. Canada and Australia have their own regulatory authorities, i.e., Health Canada and Therapeutic Goods Administration (TGA), respectively, which ensure that SaMD meets the specified safety, effectiveness, and performance level, and this evaluation mainly includes assessment at various levels, ranging from data integrity and security to clinical trials and ongoing monitoring [45,47]. While the FDA is more focused on hard factors such as algorithm performance and adaptability [43], the European Union has also included the ethical aspects of AI regulation [48]. The regulatory framework for SaMD needs to be adaptive and prioritize patient safety and effectiveness [49]. There is growing recognition of the need to address health disparities outcomes in AI-based SaMD. Auditing SaMD for biased output to assess equitable outcomes across populations is a good measure to promote health equity [50]. Regulators often require comprehensive evidence and documentation for the assessment of SaMD, and there is a need for a system view focused on risk monitoring for these devices [51]. The subsequent sections discuss aspects of the regulatory requirements as prepared by the International Medical Device Regulators Forum (IMDRF) SaMD Working Group and relate them to relevant ethical issues.

Quality Management System

IMDRF defines three principles of an effective quality management system for SaMD: first, an organizational structure ensuring the safety, performance, and effectiveness of SaMD; second, lifecycle support processes that include product planning, risk management, patient safety, record control, and analysis and improvement of product; and, third, realization and use processes that are commonly used in software engineering lifecycle [52]. These quality management approaches align with internationally accepted standards of ISO 13485:2016 [53]. These principles warrant the establishment of an organizational structure that provides appropriate leadership guidance for the development, evaluation, and regulation of SaMD and also guides manufacturers to establish processes and procedures for reliable SaMD applications [54]. However, the evolving nature of AI poses unique challenges and complexities associated with SaMD that may not be fully addressed by current guidelines [55]. The effectiveness of a quality management system to ensure the safety and performance of SaMD is entirely dependent on proper implementation, adherence, continuous monitoring, and evaluation to ensure compliance with the system to address any issue that may arise [56]. Moreover, implementing a quality management system can be costly and time-consuming and requires significant resources, and this might limit the small companies to comply with these requirements [57]. A quality management system ensures beneficence and non-maleficence by ensuring the safety of patients and promoting their well-being. It ensures the autonomy of the patient by providing comprehensive instructions about the device and helping in making informed decisions and by promoting consistency and reliability - it builds trust in the user.

Risk Assessment

Assessing the risk of an SaMD is of foremost importance in complying with the principles of medical ethics. Risk assessment refers to the process of evaluating and analyzing potential risks associated with the use of SaMD. It involves the identification of hazards, assessing the likelihood of harm and severity, and employing measures to mitigate these risks [58]. A thorough risk assessment analysis is key to finding and addressing potential risks associated with SaMD before it gets deployed in a clinical setting [51]. Risk assessment allows the regulatory bodies to categorize the SaMD into appropriate groups, from low risk to high risk, thus requiring controls accordingly. In this way, risk assessment helps in gaining trust and user confidence as it provides evidence of the safety and effectiveness of software [59]. ISO 14971:2019 deals with the aspect of risk assessment of medical devices in greater detail and provides guidelines for manufacturers in the identification and elimination of risk by using proper controls [60]. IEC/TR 80002-1 is a technical document that describes a systematic approach to risk management and serves as a guideline for developers and regulatory bodies working in the domain of SaMD [61]. One of the major drawbacks of the current risk assessment framework is its inability to provide clarity about the extent to which the development of AI algorithms is influenced by sectoral interests [62]. This can lead to potential conflicts of interest and biases and interfere with the safety of SaMD. Besides that, the narrow focus of the risk assessment process on intended use ignores the details regarding harmful consequences of potential unintended use [55]. There is also no mechanism to compare two similar algorithms directly that assesses relative safety [54], as it hinders the informed decision-making process in choosing between two devices. ISO 62304:2006 [63] lays down the requirements for the life cycle of medical device software while aligning with the principle of the quality management system and risk assessment framework. Together, these international standards try to address the major issues that arise due to direct human-computer interaction and sharing of data and minimize the risk of harm, thus enabling SaMD to achieve the goals of medical ethics.

Cybersecurity Evaluation

AI-enabled SaMD requires a huge amount of patient data for training purposes. This gives rise to questions of data security, data privacy, and data ownership. The Health Insurance Portability and Accountability Act (HIPAA) plays a significant role in this regard and sets standards for the protection of sensitive patient health information (protected health information or PHI) [64]. HIPAA ensures that the privacy and security of patient data are untouched while using AI-based systems in healthcare. Whenever AI is involved in a clinical setting, the relevant organization must comply with the regulations set by HIPAA to ensure that data are handled per privacy standards and kept securely [65]. One of the key concepts in HIPAA is the process of de-identification. This allows researchers to use patient data without their consent as long as the data is de-identified and does not reveal any personal information. De-identification involves either removing or altering specific identifiers, thus protecting patient privacy while allowing the researchers to use data for training and analysis [66]. HIPAA binds covered entities to conduct a risk assessment and implement appropriate physical, technical, and administrative safeguards to protect PHI. This includes access control measures, encryption of data, audit trails, employee training, and incident response plans [67-70]. HIPAA requires the covered entities to maintain business associate agreements while working with third parties having access to PHI. This agreement outlines the obligations of business associates in protecting patient data and ensuring HIPAA compliance as well as cybersecurity measures [67,71,72]. Even though HIPAA does provide guidelines for cybersecurity measures, it does not outline a comprehensive cybersecurity framework and leaves it to the relevant authority to ensure that all requirements for cybersecurity are met. In the European Union, under the General Data Protection Regulation (GDPR), patients have the full right to their data and decide where and how it can be used.

Adoption of artificial intelligence in healthcare

**Figure 2:**
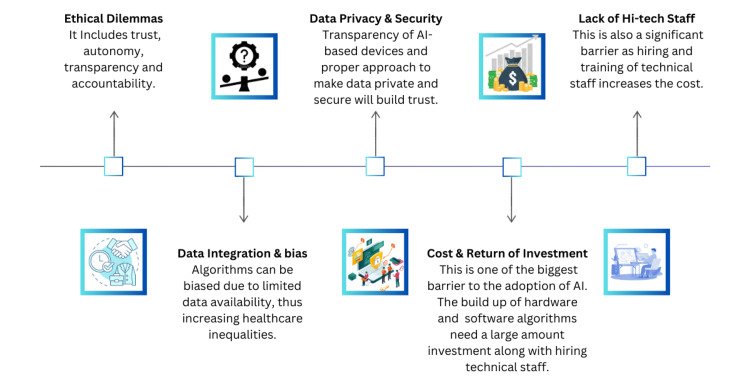
Barriers to adoption of artificial intelligence in healthcare The figure was created by the authors.

To leverage the full potential of AI-based devices in clinical settings, addressing the barriers to adoption is the first step (Figure 2). This can be achieved by understanding the perspective of healthcare professionals and taking measures to address the issues of trust, usability, and workflow integration [73]. Workflow integration must be preceded by a thorough evaluation of ethical implications, data privacy and security, and potential biases of AI [74]. Regulatory authorities, AI researchers, manufacturers, and healthcare organizations should collaborate to address the issues preventing the full adoption of AI technologies in healthcare settings. The collaborators must work to develop normative standards and evaluation guidelines ensuring the safety, quality, ethical aspects, and transparency of AI-based devices as well as supporting research and innovation in the field by resource allocation [74,75]. The NASSS (Non-adoption, abandonment, Scale-up, Spread, and Sustainability) framework can also guide the AI implementation in healthcare by identifying the factors facilitating or hindering successful AI implementation [76]. Collaboration between stakeholders and investment from the government is identified as an important factor to build a more efficient and effective healthcare system based on AI-based technologies.

One of the major hindrances in the adoption of AI-based technologies by healthcare providers is the “Black Box'' nature of AI algorithms. The Black Box nature of AI refers to the lack of transparency and interpretability of AI’s decision-making process [77,78]. As clinicians do not know about the factors contributing to a particular prediction made by an AI algorithm, they are skeptical to base their trust on these models [79]. As the model is not explainable and transparent, the question of accountability and liability arise whenever adverse outcomes occur. With explainable AI, you can develop better inventory management, interpret designs easily, and reduce costs with high performance [80]. In the healthcare system, AI-based devices and applications are also constrained by a lack of scientific evidence and adopters' acceptance and trust. The role of local champions in overcoming these barriers is crucial [76]. Extensive research is needed in this field to make AI more trustworthy and acceptable in clinical disciplines. One approach that can be taken to make the AI algorithm more interpretable is the use of the Lorenz-Shapley model, which combines Shapley values with a statistical normalization based on Lorenz zonoids and offers a framework to make the AI model interpretable and explainable [81].

Limitations

This article has several limitations. First of all, an unconventional approach to the literature search was taken. The traditional PRISMA guideline approach for systematic review with keywords “Artificial intelligence”, “Machine Learning”, “Deep Learning”, “Software as Medical device”, “SaMD”, “Ethics”, “Ethical”, “Regulatory”, “Regulation” did not yield enough literature to comprehensively cover the topic. Therefore, a flexible and iterative process was employed for literature search that leveraged the interconnected nature of scholarly articles. Key terms were used to identify the papers in PubMed and the Institute of Electrical and Electronics Engineers (IEEE) databases in the domain of AI ethics, SaMD regulations, and the regulatory process of SaMD. The process of snowballing was employed where relevant articles were identified by a constant exploration of the literature pool. Another limitation of this study is the lack of a comprehensive overview of the current regulatory framework of SaMD. Only those aspects of SaMD regulations were discussed that were directly relevant to one of the four principles of medical ethics. Finally, the field of medical ethics has gained a vast amount of new values and terms. Jobin et. al. [28] identified 11 clusters of ethical principles but for the sake of simplicity, but in this paper we only discussed the traditional principles of medical ethics and placed other values in those principles, for example, informed consent is mentioned as a part of autonomy while discussing the ethical principles mentioned above [28].

## Conclusions

AI has significant potential to be leveraged for better healthcare provision by early detection and reduced time of care delivery. The existing regulatory approval process of AI-based SaMD, although successfully evaluated, fulfills the technical requirements of AI-based SaMD but the ethical questions related to their usage are still an area of research. The ethical questions that are still in research and in need of proper guidelines are data privacy, data security, and data ownership. The ethical issues arising from the use of AI technologies can be addressed by a robust and systematic SaMD approval framework developed by the collaboration between AI researchers, healthcare professionals, social scientists, and regulators. The existing regulatory frameworks are neither holistic in their approach nor universal. An extensive regulatory process encompassing all aspects of medical ethics as well as software development and clinical performance of these devices must be developed and made public. More such steps should be taken to involve the public in the decision-making process regarding these technologies as they are going to be end users for them. An approach combining both soft and hard aspects of AI-based SaMD will result in an explosion of its adoption in clinical settings and help in achieving universal healthcare.
